# A Hybrid BCI Based on SSVEP and EOG for Robotic Arm Control

**DOI:** 10.3389/fnbot.2020.583641

**Published:** 2020-11-20

**Authors:** Yuanlu Zhu, Ying Li, Jinling Lu, Pengcheng Li

**Affiliations:** ^1^Wuhan National Laboratory for Optoelectronics, Britton Chance Center of Biomedical Photonics, Huazhong University of Science and Technology, Wuhan, China; ^2^MoE Key Laboratory for Biomedical Photonics, School of Engineering Sciences, Huazhong University of Science and Technology, Wuhan, China; ^3^Huazhong University of Science and Technology-Suzhou Institute for Brainsmatics, Suzhou, China

**Keywords:** hybrid brain-computer interface (BCI), electrooculography (EOG), robotic arm control, steady-state visual evoked potential (SSVEP), information transfer rates (ITR)

## Abstract

Brain-computer interface (BCI) for robotic arm control has been studied to improve the life quality of people with severe motor disabilities. There are still challenges for robotic arm control in accomplishing a complex task with a series of actions. An efficient switch and a timely cancel command are helpful in the application of robotic arm. Based on the above, we proposed an asynchronous hybrid BCI in this study. The basic control of a robotic arm with six degrees of freedom was a steady-state visual evoked potential (SSVEP) based BCI with fifteen target classes. We designed an EOG-based switch which used a triple blink to either activate or deactivate the flash of SSVEP-based BCI. Stopping flash in the idle state can help to reduce visual fatigue and false activation rate (FAR). Additionally, users were allowed to cancel the current command simply by a wink in the feedback phase to avoid executing the incorrect command. Fifteen subjects participated and completed the experiments. The cue-based experiment obtained an average accuracy of 92.09%, and the information transfer rates (ITR) resulted in 35.98 bits/min. The mean FAR of the switch was 0.01/min. Furthermore, all subjects succeeded in asynchronously operating the robotic arm to grasp, lift, and move a target object from the initial position to a specific location. The results indicated the feasibility of the combination of EOG and SSVEP signals and the flexibility of EOG signal in BCI to complete a complicated task of robotic arm control.

## Introduction

Brain-computer interfaces (BCIs) are designed as a bridge to construct direct communication between the brain and external devices without relying on normal peripheral nerves and muscle tissue (Wolpaw et al., 2000). BCIs aim to provide people with severe motor disabilities an alternative to communicate and control external devices. Robotic arm control is one of the popular applications of BCI. Many studies have attempted to realize BCI for robotic arm control to improve the life quality of people with motor impairment (Pfurtscheller et al., 2010b; Gao et al., 2017; Khan and Hong, 2017). Considering the practical use of people with motor disabilities, the system design should focus more on the accuracy of command execution and the convenience of operation.

Electroencephalography (EEG) is one of the most widely used non-invasive BCI for its low cost, portability and high temporal resolution. Several types of physiological activation are usually chosen to generate the output commands of the EEG-based BCI, such as motor imagery (MI) (Wolpaw et al., 1991), P300 (Farwell and Donchin, 1988), and steady-state visual evoked potential (SSVEP) (Cheng et al., 2002). Single modality which uses only one type of input signal usually has its own limitation in the number of commands and the classification accuracy. To promote the application of BCIs, several researches employed multiple modalities to improve the performance of the system by combing the advantages of different modalities, which is named as hybrid BCI (Pfurtscheller et al., 2010a).

As for the convenience of the system operation, electrooculography (EOG) is a good choice for its easy to execute and detect. EOG is the depolarization and hyperpolarization between retina and cornea caused by different eye movements, forming a potential difference between retina and cornea whose amplitude is larger than that of EEG and background physiological signals. Therefore, EOG can be easily and accurately detected using a few of electrodes around eyes. Compared to the conventional hybrid BCI most of which utilized the multiple types of EEG signals, the combination of EEG and EOG signals to construct a hybrid BCI can reduce the workload of users and makes the operation more convenient. The eye movements often used are blinking, winking, frowning, and gazing. Several studies used EOG in BCI to reflect the intention of subjects and to transmit commands to external devices. Nakanishi and Mitsukura proposed a wheelchair control system by using the voluntary eye blink (Nakanishi and Mitsukura, 2013). Ma et al. introduced a multithreshold EOG detection method and combined the EOG and P300 for robot control which used different eye movements to obtain the control commands and turn the stimulus on and off to enhance the performance of the system (Ma et al., 2015). He et al. proposed a hybrid BCI based on MI and EOG signals to operate a web browser (He et al., 2017). Wang et al. combined MI, P300, and EOG signals to asynchronously control a wheelchair (Wang et al., 2014). Huang et al. used EOG for button selection, MI for directional control, and combined computer vision for the control of an integrated wheelchair robotic arm system (Huang et al., 2019). Tan et al. applied autoencoder-based transfer learning in hybrid BCI for rehabilitation robot which composed of MI-based rehabilitation action, SSVEP-based menu selection, and EOG-based operation confirmation of cancellation (Tan et al., 2019).

To achieve asynchronous SSVEP-based BCI system, several studies distinguished the control state from idle state by using threshold criteria during the stimulus flashing (Ortner et al., 2011; Pan et al., 2013; Zhou et al., 2020). When the stimuli kept flashing since the start of experiments including control and idle states, asynchronous BCI used threshold criteria were susceptible to be incorrect activated due to the implicit attention to the flicker stimuli. Considering the effect of stimulus flicker on asynchronous detection, other studies applied a switch to activate or deactivate the stimulus flicker (Pfurtscheller et al., 2010b; Gao et al., 2017; Li et al., 2018). Pfurtscheller et al. used sequential MI-based brain switch to turn on or off the SSVEP-based BCI (Pfurtscheller et al., 2010b). The low classification performance of MI lead to a FAR with 1.46 per minute. Given the high signal-to-noise ratio (SNR) of EOG signals, Li et al. applied a single blink synchronized with a random flashing button as the switch of wheel chair control (Li et al., 2018). They used two consecutive intended blinks as a start command with no false option occurred in static state and an intended blink as a stop command with a FAR of 0.18 per minute in the motion state. Due to the switch detection based on the synchronization of the flicker button and a single blink, the button was required to flash in the idle state.

In present study, to further decrease the FAR of asynchronous SSVEP-based BCI, we designed an EOG-based switch with no need for stimulus in idle state and combined the switch with a timely cancel command to effectively control a robotic arm. Through using the EOG-based switch to activate and deactivate the flicker stimuli, there was no need for extern stimulus in idle state which decreased visual fatigue caused by flashing and was more in line with perception of idle state. The FAR of the proposed asynchronous SSVEP-based BCI related only to the detection accuracy of EOG signals. Due to the high SNR of EOG signals, the detection of EOG is more accurately. We selected the triple blink as the EOG-based switch due to its ease of completion and low probability of occurrence in normal physiological situation. Moreover, we designed a cancel command based on a wink to make the subject be able to cancel the execution of a current command as needed. The control commands of the robotic arm were obtained by a SSVEP-based BCI, considering that SSVEP has gained a lot of attention in BCI for the reason of less training, high classification accuracy and information transfer rates (ITR). Fifteen buttons consist of the graphical user interface (GUI) of the SSVEP-based BCI, subjects were asked to focus on one of the fifteen buttons in a flash cycle to transmit corresponding control commands to the robotic arm. Given the relatively large number of stimuli, the default setting is to execute the feedback command, which was only canceled when the feedback stage recognized a wink from subject to helped control the robotic arm to complete the action more effectively. The algorithm of detecting SSVEP used was filter bank canonical correlation analysis (FBCCA) method (Chen et al., 2015a) which made use of the information in harmonic frequencies to improve classification accuracy. A multi-threshold method (Ma et al., 2015) was adopted to detect different eye movement waveforms. The experimental results showed the feasibility of the proposed system and the ability to complete a complicated task with a series of actions through the combination of EOG and SSVEP signals for robotic arm control.

## Materials and Methods

### Subjects

Fifteen healthy volunteers (8 female; age 24.9 ± 2.5 years) with normal or corrected-to-normal vision participated in the experiments. Eleven subjects conducted two online experiments to evaluate the performance of the proposed hybrid EOG-SSVEP-based BCI. Six subjects conducted comparable experiments to evaluate the effectiveness of using EOG for command cancellation. All subjects are undergraduate and graduate students, and three of them have some experience with MI-based BCI experiments, while others are naive to BCI experiments. Before the experiments, each subject read and signed an informed consent form approved by the Human Subjects Institutional Review Board of Huazhong University of Science and Technology. Subjects obtained a small compensation for participating in the experiments.

### Data Acquisition System

**Figure 1:**
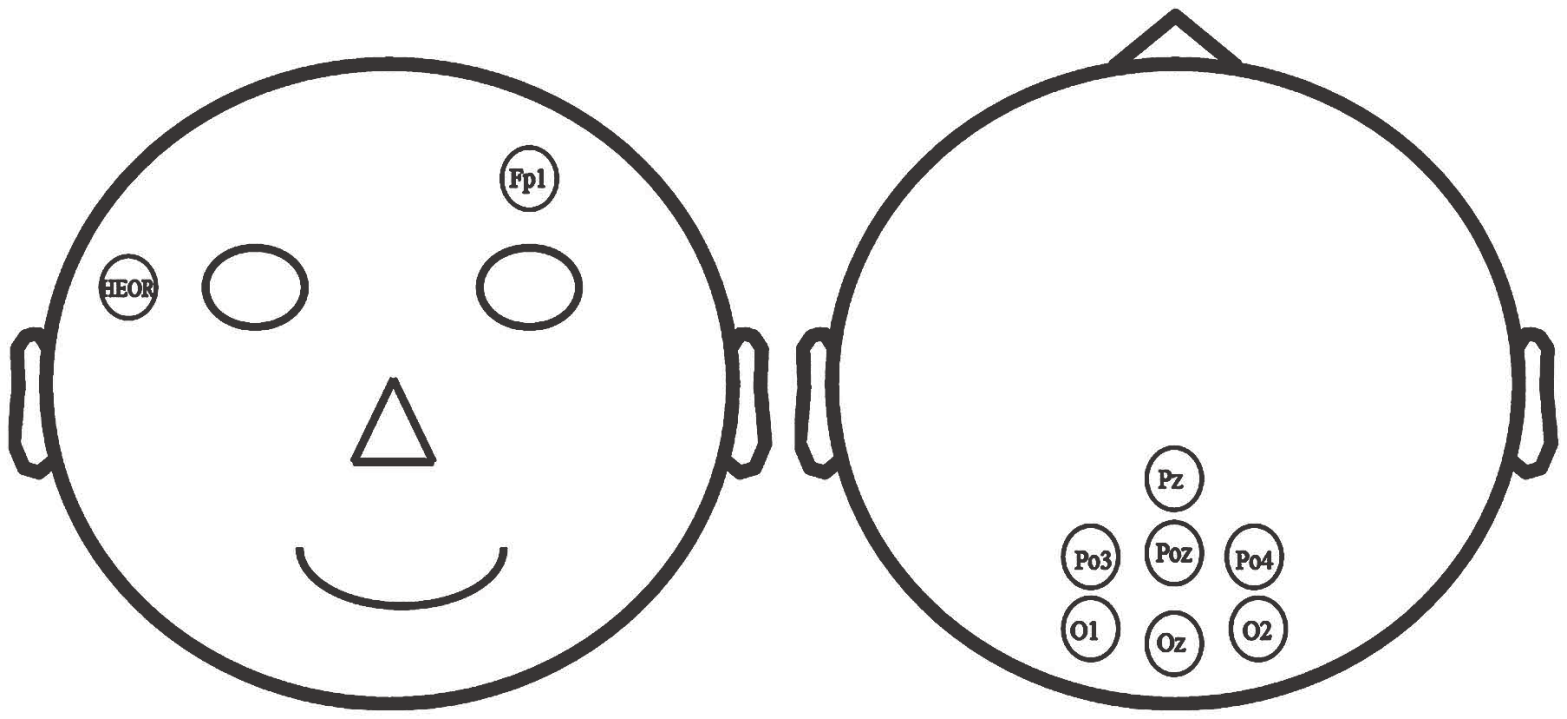
Location of nine electrodes for EEG recording.

In this study, EEG and EOG signals were recorded at sampling rate of 250 Hz with high-pass and low-pass filters of 0.1 and 250 Hz using a multichannel EEG system from Brain Products (BrainAmp, Germany). A total of nine electrodes, HEOR, Fp1, Pz, PO3, POz, PO4, O1, Oz, and O2 were placed according to the International 10–20 system (see Figure 1). Electrodes Pz, PO3, POz, PO4, O1, Oz, and O2 were used to collect SSVEP-based EEG raw signal, and the electrodes HEOR and Fp1 were selected to record the EOG signal. The electrode on the forehead (AFz) was used as ground and the reference electrode was positioned on the vertex (Cz). All electrodes impedances were maintained below 10 KΩ.

The visual stimuli were presented on a 23.8-inch LCD screen with a resolution of 1,650 × 1,080 pixels. The refresh rate of the screen was 60 Hz. All subjects were arranged to seat in a comfortable chair in front of the visual stimulus computer at a distance of ~70 cm in a quiet room. The visual angle between the robotic arm and the monitor was 45°. This arrangement allowed subjects to look at both the monitor and the movement of the robotic arm.

### GUI

**Figure 2:**
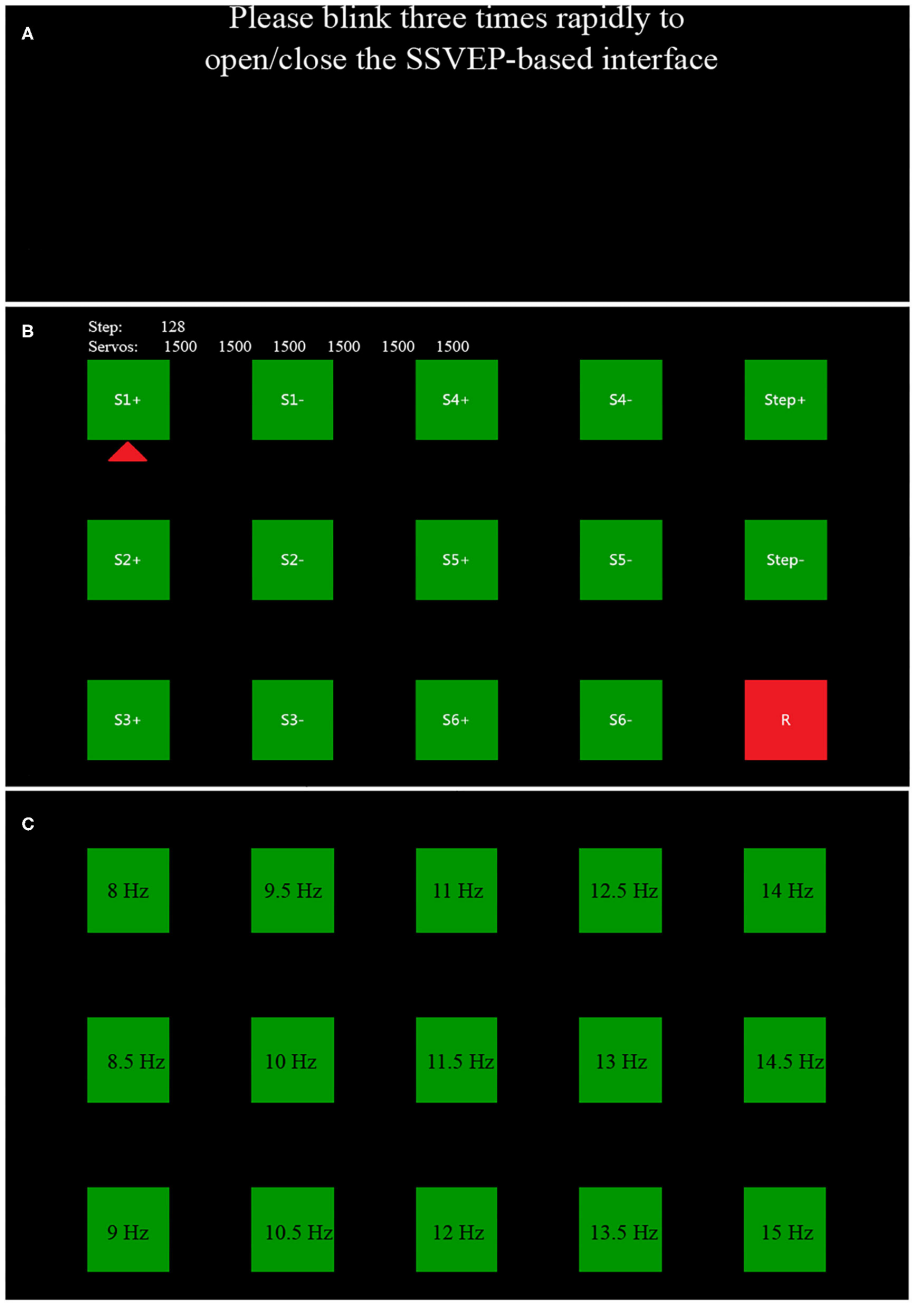
The GUI of EOG-based switch **(A)** and SSVEP-based BCI **(B)**. In **(C)**, a 3 × 5 flashing stimulus matrixes labeled with different stimulus frequency represents a total of 15 commands for the robotic arm control.

The GUI was designed to ensure the effective and accurate control and operation of the robotic arm. As illustrated in Figure 2, the GUI was composed of two sessions: the switch interface which displayed “Please blink three times rapidly to open/close the SSVEP-based interface” in the center of the screen to prompt the subjects to use the EOG-based switch, and the SSVEP-based interface consisted of a 3 × 5 flashing stimulus matrix representing 15 commands which were designed to control the robotic arm for the grasp and move actions. Visual flashing buttons of the SSVEP-based interface were presented using a sampled sinusoidal stimulation method (Manyakov et al., 2013; Chen et al., 2014). The size of each button was 150 × 150 pixels. All buttons flashed between green and blue under black background to reduce visual fatigue (Takano et al., 2009; Chen et al., 2017; Floriano et al., 2018). The horizontal and vertical distance of each adjacent buttons was 150 pixels. The range of the stimulus frequency for the fifteen visual flashing buttons in the proposed study was chosen from 8 to 15 Hz with an interval of 0.5 Hz because of its relatively high response in their corresponding SSVEP signal (Chen et al., 2018). The stimulus paradigm of the BCI was realized by using the Psychophysics Toolbox Version 3 (Brainard, 1997) on MATLAB (MathWork, Inc).

As shown in Figure 2B, there were two lines of text at the top of the SSVEP-based interface to assist the subjects in getting the real-time status of the robotic arm. The first line of text indicated the current programmed moving step of the robotic arm, and the second line of text displayed the corresponding configured position of each axis of the robotic arm. The left four columns of buttons corresponded to different directions of the movements of the robotic arm. And the robotic arm had a total of six axis (S1, S2, S3, S4, S5, and S6). For a specific direction, “S1” represented the rotation of the robotic arm in the x-y plane, and “S2,” “S3,” and “S4” allowed the robotic arm to move to different degrees along the z-axis. “S5” was used to rotate the claws. “S6” drove the robotic arm to clamp or loosen. Among them, “S1+” and “S1–” indicated the opposite direction, respectively, and others were the same. In order to make an effective operation, two buttons (“step+” and “step–”) were added to change the moving step of the robotic arm movements in different direction. “R” was utilized to return the robotic arm to its original position. Figure 2C showed the stimulation frequency of each target.

### System Configuration Description

**Figure 3:**
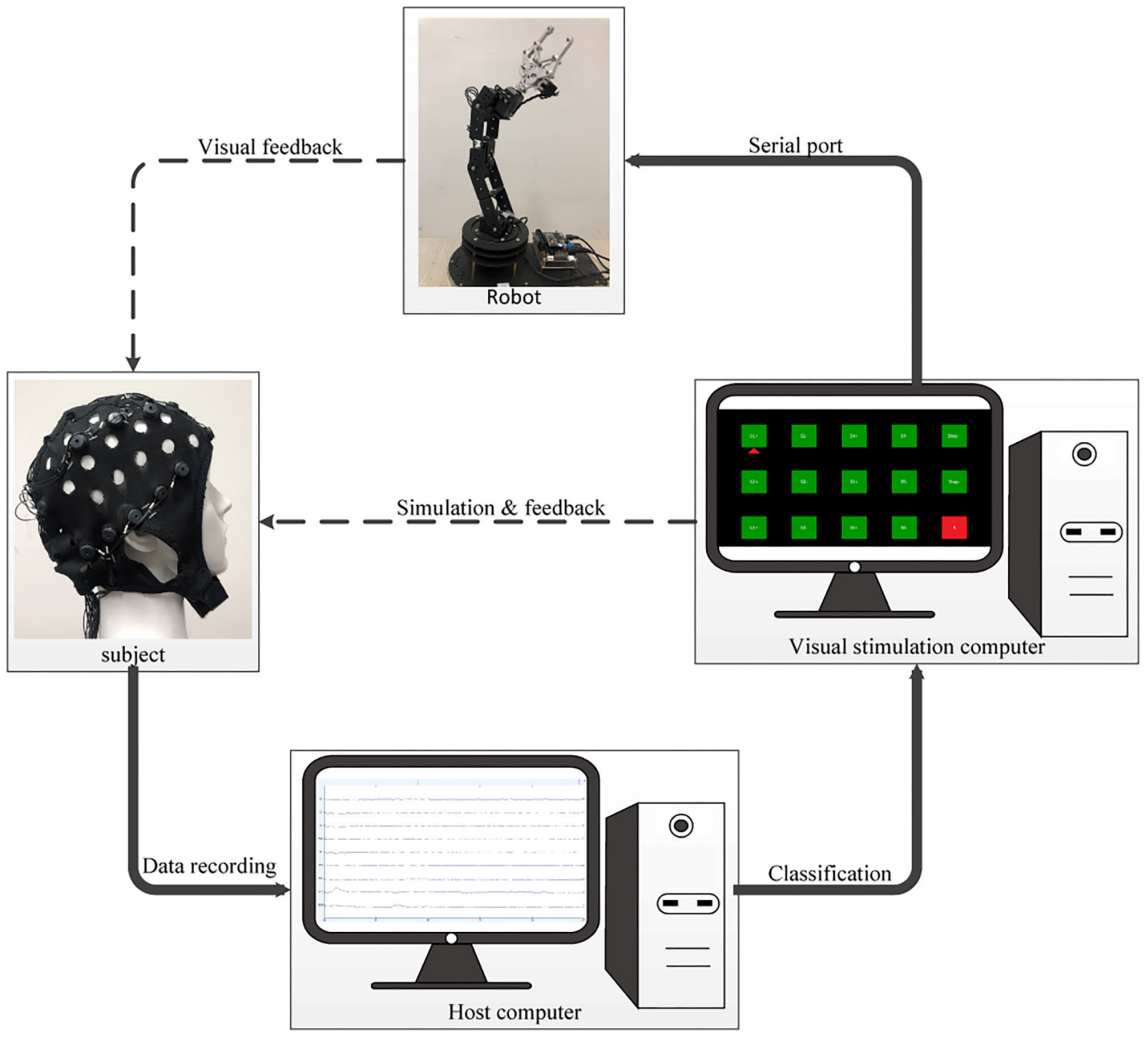
Schematic configuration of the proposed hybrid BCI for robotic arm control.

For practical use, the design of multitask makes the control and operation of the BCI more flexible and versatile. This study combined the eye movements and SSVEP to realize an asynchronous hybrid BCI. As illustrated in Figure 3, the proposed asynchronous EOG-SSVEP-based robotic arm control system mainly consisted of four hardware components: an EEG acquisition device, a visual stimulus computer, a robotic arm, and a host computer used as data online processor. The EEG signals were recorded and transmitted to the host computer with synchronous event triggers sent from the visual stimulation computer for real-time preprocessing and classification. The visual stimulation computer was not only utilized to present the stimulus paradigms and online visual feedback but also for translating relevant commands to the robotic arm via serial communication protocol. Six axis (ZX-361S) and an open source STM32 control board composed of the robotic arm which was able to be directly and easily controlled by the SSVEP-based interface through serial port. The manipulating angle of each axis was configured in the range of 0° to 180°, and the rotation speed of each axis could be adjusted according to the moving step which was set by the subjects.

**Figure 4:**
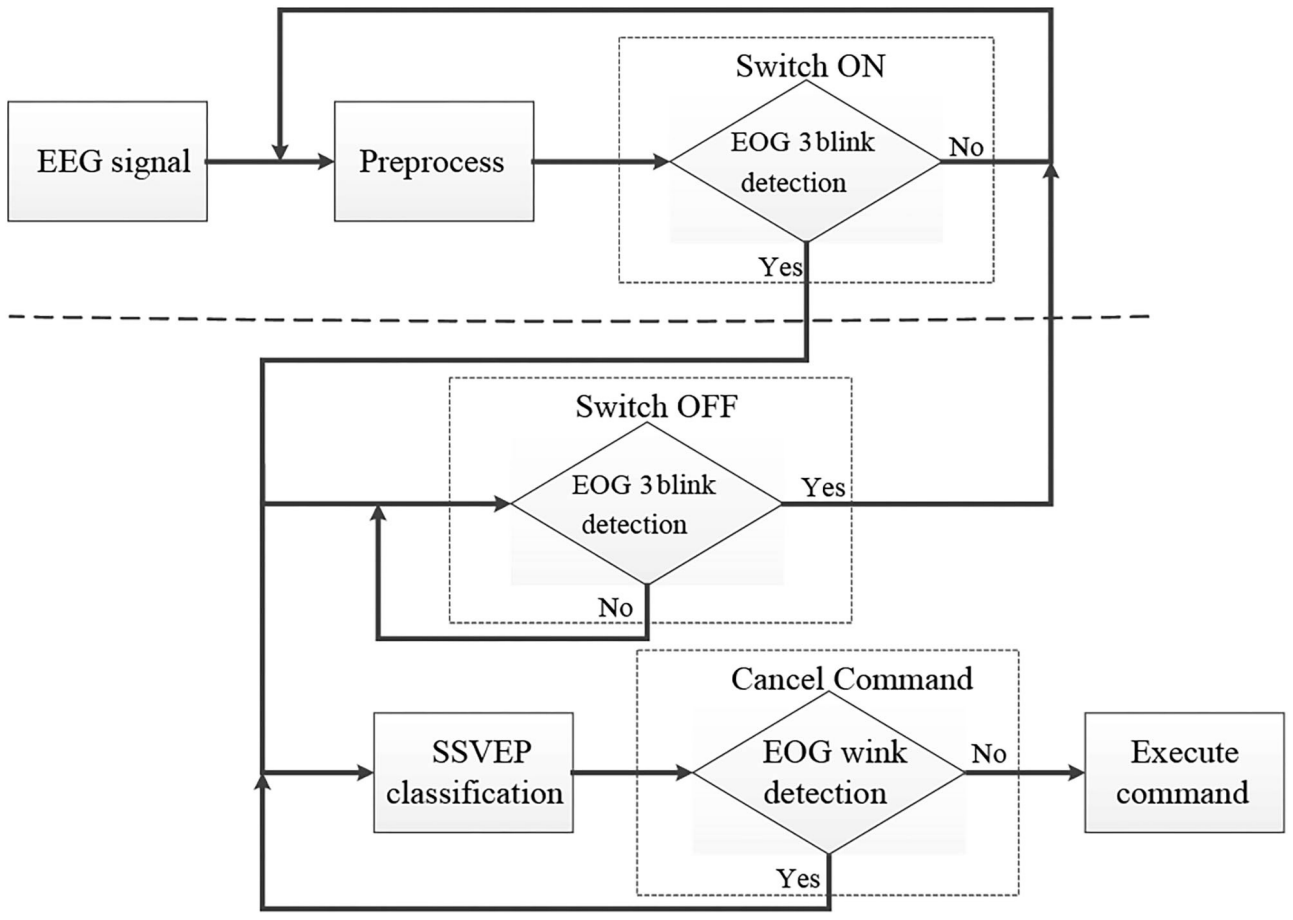
Flowchart of the proposed system which consists of an SSVEP-based BCI with EOG-based switch.

The system flowchart can be seen in Figure 4. After the start of the experiment, EEG data recorded from the subject are first preprocessed to remove the baseline drift and the influence of the environment. And then the SSVEP interface can be activated only when a triple blink from the subject is detected by the system. Otherwise the system will maintain in the switch interface. To effectively detect EOG signal when subjects blink three times rapidly, a calibration process was conducted before online experiment to determine the appropriate online threshold for each subject. When the SSVEP-based interface is activated, the SSVEP signal and the triple blink are detected in parallel. Subjects are allowed to blink three times rapidly when they hope to switch off the flash of the buttons and return to the EOG-based switch interface. If no triple blink is detected, the classification of the SSVEP signal will be transmitted to the visual stimulus computer as a feedback to the subject. And a robotic control command corresponds to the specific classification result. Once the subject wants to cancel the command sending to the robotic arm, he or she is asked to execute a wink after the occurrence of the feedback. If no wink is detected at the feedback phase, the robotic arm will execute the relevant command and then another flashing cycle begin to generate another new command.

### Data Processing and Detection Algorithm

In this study, the EEG and EOG data were processed and fed back in real time. The recording data were firstly preprocessed to minimize the impact of external environment and motion artifacts. The 50 Hz interference of power supply was eliminated by a notch filter. The EEG signals were then re-referenced to the arithmetic average across all recording channels. The detection algorithm was mainly composed of two parallel parts: EOG data analysis and SSVEP data analysis.

#### EOG Data Analysis

The proposed asynchronous hybrid BCI allowed subjects to decide when to start and stop the control of the system by an EOG-based switch which was designed by the detection of a triple blink from the Fp1 channel. The detection is conducted every 100 ms from the beginning of the experiment. For real-time analysis, we use a sliding window with a length of 1,200 ms with an interval of 100 ms. Furthermore, a wink was used to cancel the command in feedback phase, and the detection for wink is based on a segment data with a length of 2000 ms which contained the feedback and remind phase from the HEOR channel. The length of data is set in consideration of the reaction time of subjects. The detection method for the triple blink and wink is based on a multithreshold method descripted in Ma et al. (2015). For each detection, first, a segment data is extracted according to the window set for different eye movements. Then, on the purpose of removing physiological and environmental noise, the extracted data are bandpass filtered within the range of 0.1 to 15 Hz, after that the first-order difference operation is employed to get features of the eye movements as follows:

$$\begin{array}{l} f^{\prime }\left(n\right)\;=\;f\left(n\right)-f\left(n-1\right) \end{array}$$

**Figure 5:**
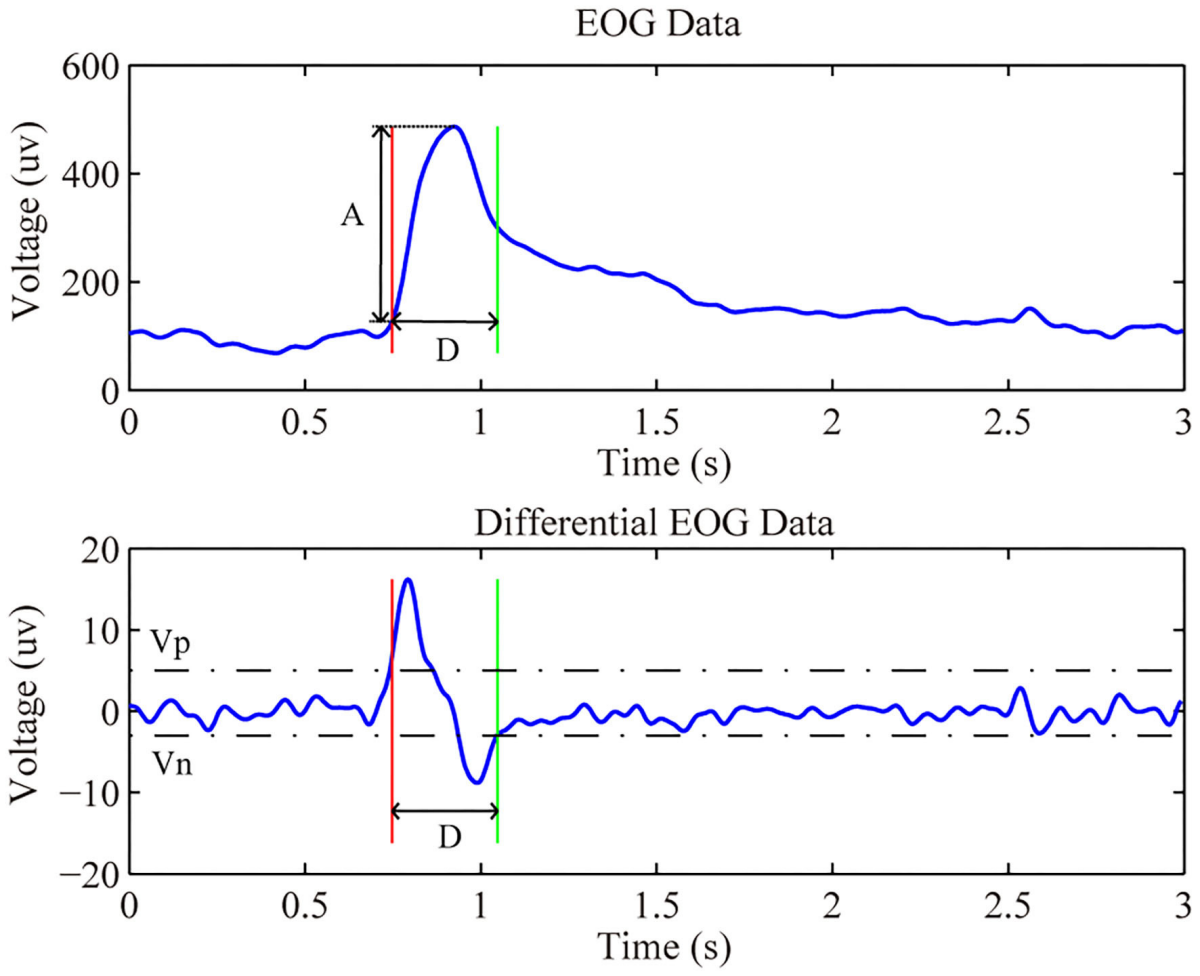
The raw and differential EOG data of a wink.

Where *n* is the sampled points, *f(n)* is the relevant original value, and *f* ′*(n)* refers to the differential value of the original data at point *n*. There are several features abstracted from the differentiated waveform for later analysis, e.g., the maximum peak value, the minimum peak value, the maximum amplitude, and the duration of the eye blink. Figure 5 showed the raw and differential EOG data from channel HEOR in a trial that prompted the subject to wink during the calibration session. The differential EOG data of a wink contained a positive and negative wave, and its amplitude was much larger than the fluctuation of the EOG when no wink is performed. In order to extract the signal from a wink, we need to set a minimum value (Vp) for a positive wave. The signal is considered to be a positive wave when its voltage is greater than the Vp. Likewise, we need to set a maximum value (Vn) for the negative wave. The signal is considered to be a negative wave when its voltage is less than the Vn. When a positive wave followed by a negative wave is satisfied, to avoid recognizing the rest period signal as a wink, it is also necessary to satisfy that the amplitude of the original EOG (A) in the time period (D) from the beginning of the positive wave to the end of the negative wave is greater than the amplitude (Amp) of the initial setting, and the length of the time period (D) needs to be greater than the limited minimum duration (Dmin) and less than the limited maximum duration (Dmax) of the initial setting, then it is considered as a wink. The principle of a successful eye blink detection are as follows:

$$\text{i }=\;\{\begin{array}{cc} \;1, & \text{if A}\geq \text{Amp},\;{\text{D}}_{\min}\;\leq \text{D }\leq \;{\text{D}}_{\max}\\ 0, & \text{otherwise} \end{array}$$

Where *i* represents the detection result of a wink. If the features of the EOG waveform satisfy all condition, *i* is equal to 1 which means a successful wink detection, otherwise *i* is equal to 0 manifests no intentional wink was detected. Thus, the recognition of a wink requires the initial setting of five thresholds, including Vp, Vn, Amp, Dmin, and Dmax. Since the duration of a wink does not vary much between subjects, Dmin and Dmax were set to 0.1 and 0.6 s for all subjects, respectively. The other three thresholds are influenced by the way each subject winks, so it is necessary to set specific thresholds for each subject in order to accurately identify the wink of each subject.

**Figure 6:**
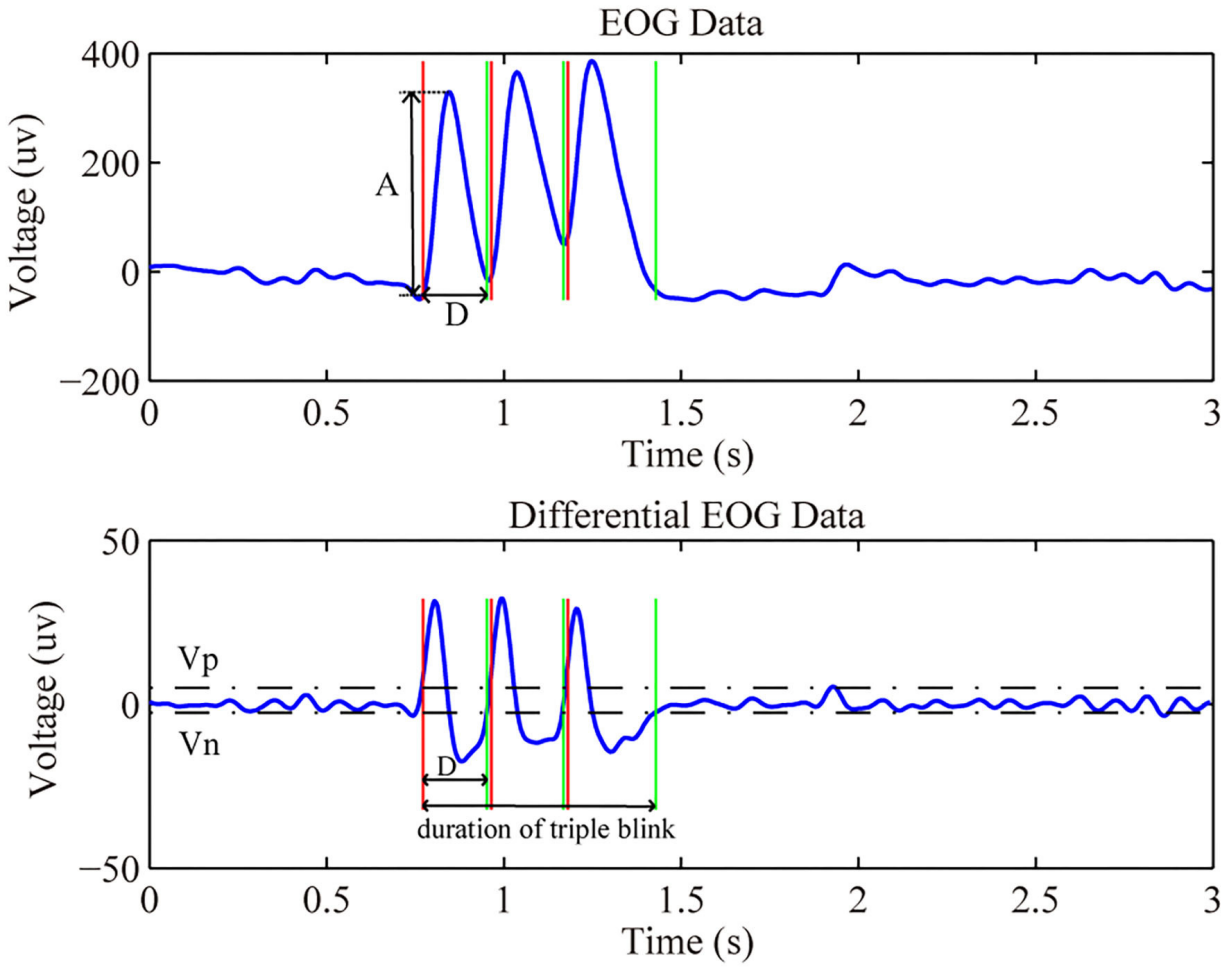
The raw and differential EOG data of triple blink.

The basic algorithm for identifying triple blink is the same as the detection of a wink, except that it is considered as the triple blink only when three consecutive blinks are recognized within a limited time window length which is set to 1,200 ms. The Figure 6 showed the raw and differential EOG data from channel Fp1 in a trial that prompted the subject to conduct triple blink during the calibration session. Therefore, to effective identify the intentional eye movements in online experiments, a calibration process was asked to conduct for each subject first to obtain the thresholds required in the detection algorithm mentioned above.

#### SSVEP Data Analysis

In this study, the flash of the SSVEP-based interface was activated by the EOG-based switch. Once activated, the data epochs were extracted according to the event triggers for subsequent classification and the control of the robotic arm. Previous studies have shown that SSVEP induced by periodic visual stimulus contains brain response at the stimulus frequency and its harmonic and sub-harmonic frequencies (Herrmann, 2001). This study adopted the filter bank canonical correlation analysis (FBCCA) method (Chen et al., 2015a,b) which can effectively utilize information in harmonic frequencies to enhance the detection of SSVEP. FBCCA method is mainly composed of three steps. First, a filter bank which consists of several bandpass filters decomposes the SSVEP data epochs into sub-band components. Second, an canonical correlation analysis (CCA) approach which has been widely adopted in BCI for SSVEP detection (Bin et al., 2009) is applied to get the correlation between sub-band components and predefined sinusoidal reference signals. Last, appropriate feature vectors are calculated for the target identification. In this study, we used CCA and FBCCA methods for the classification of SSVEP signals, and applied paired *t*-test for statistical analysis to evaluate the performance of CCA and FBCCA methods.

Classification accuracy and ITR were used to evaluate the performance of the proposed system. ITR was calculated according to the follow equation (Wolpaw et al., 2002):

$$ITR\;=\;\frac{60}{T}\left(log_{2}N+\text{P}log_{\text{2}}\text{P}+\left(1-\text{P}\right)log_{2}[\frac{1-P}{N-1}]\right)$$

Where T is the time it takes to output a command, including the time of the gaze shift, stimulus flicker, and feedback phase, *N* is the total number of targets (*N* = 15), and *P* is the classification accuracy.

### Calibration Process

For the purpose of effectively detecting the eye movements in real time, a calibration process is acquired to determine thresholds of different eye movements for each subject before online experiments. Appropriate detection thresholds allow the system to have short response time and high accuracy which make the system more reliable and flexible. During the calibration process, the paradigm of a trial consists of three parts. First, a fixation cross appears in the center of the visual stimulation screen for 1 s to prompt the subject to get ready for the task, and then the screen shows a text of “triple blink” or “wink” for 4 s to remind the subject to blink three times rapidly or wink in task period, after that 1 s black screen is displayed as a rest period. A total of 20 trials for triple blink and 20 trials for wink were designed for each subject to collect the datasets for the calculation of respective detection thresholds and to train subjects to be familiar with the eye movements simultaneously.

For offline processing, the recording data for triple blink and wink were extracted from all trials for thresholds calculation. We used the detection algorithm described in the EOG data analysis to get the specific thresholds of triple blink and wink for each subject. Specifically, we calculated the first-order difference of the bandpass filtered data. In order to identify eye movements in task period, a predefined experiential threshold was used for the sampled data. The features of the eye movement waveforms were then computed. We removed the features of unqualified samples which only contained the motion artifacts but did not eye movements. The thresholds of online experiments was decided by the remaining features. The main thresholds to be obtained were Vp, Vn, and Amp.

### Experiments

Before the online experiments, a calibration session mentioned above was carried out to determine appropriate online thresholds for each subject. Then two online experiments were performed using our asynchronous hybrid BCI system. One was a cue-based experiment, and the other was a self-paced operation of the robotic arm to conduct with a complicated task.

#### The Cue-Based Experiment

This experiment contained eight blocks which was designed to assess the performance of the proposed system and train subjects to be familiar with the procedures of the system control. Each block consisted of two parts: the operation of the EOG-based switch and the gaze of the cue-based SSVEP interface for robotic arm control. Besides, in order to evaluate the capability of the use of winking to cancel the command in feedback phase, before the flicker of SSVEP-based interface, the clue given in GUI requested the subjects to wink or not to wink in the feedback phase of the current block. The clue indicating the request of a wink appeared every 2 blocks. A total of 4 blocks contained the data of winks. At the beginning of each block, the screen displayed the switch interface to prompt subjects to blink three times rapidly to activate the flash of the cue-based SSVEP interface. And then a cue with a red triangle appeared under one of the fifteen buttons with a pseudo-random order. There were a total of fifteen trials for the test of the SSVEP-based interface. Each trial with a duration of 5.5 s consisted of a remind phase for 0.6 s and a stimulation phase for 3 s and a feedback phase for 1.9 s. Subjects were asked to shift their gaze to the button indicated by the cue in the remind phase, and focus on the button in the stimulation phase to ignore the influence of other buttons as much as possible. In the feedback phase, a button was marked in red color according to the real-time classification. Subjects decided whether to use a wink to cancel the command based on the clue given in the start of the GUI. If there was no wink in the feedback phase, the corresponding command was transmitted to the robotic arm for motion control. When all buttons flickered once, the switch interface was appeared to remind subjects to use a triple blink to deactivate the flash of the SSVEP-based interface and take a break before the next block.

#### Asynchronous Robotic Arm Operation

In this experiment, the commands were selected by subjects to execute a series of sequential actions without visual cues. In order to maintain the difficulty of the experiment, the start and end location of the target object were consistent for each subject. Moreover, the robotic arm was reset to its initial position before the start of each operation. For the procedures of the experiment, in the first step, subjects used a triple blink to activate the flash of the SSVEP-based interface when they were ready to control the robotic arm. The paradigm of the SSVEP-based interface was mostly the same as the cue-based experiment except that there is no visual cues in the remind phase. Each trial started with the appearance of all stimuli in static state without visual cues, which lasted 0.6 s for attention and gaze shifting. Then the stimulus began to flicker with a duration of 3 s. Last, the screen displayed the online feedback for 1.9 s, giving the subjects a chance to decide whether he or she will cancel the corresponding command or not. A wink after the appearance of the feedback could be identified as a canceled intention. Subjects were asked to operate the robotic arm to perform a series of actions of moving, grasping, lifting, and placing by gazing the SSVEP-based interface. After placing the target object to the specific location, the reset command was used to reset the robotic arm by gazing the “R” button twice before the next operation. The task execution time was recorded by the host computer based on the synchronous event triggers. To avoid misidentification, the reset command was executed only when the “R” button was detected in two consecutive trials. During the experiment, subjects were able to use a triple blink to deactivate the flash of SSVEP-based interface whenever they needed to rest and adjust.

## Results

As for the cue-based experiment, the classification accuracy and ITR were calculated to evaluate the performance of the SSVEP-based BCI. The false activation rate (FAR) which meant the rate of false triggering (Wang et al., 2014) was used to assess the efficiency of the EOG-based switch. The FAR was calculated by dividing the number of false identifications of the triple blink during the stimulus flicker by the duration time of the cue-based experiment. The false positive rate (FPR) and true positive rate (TPR) of the wink were computed to evaluate the reliability of the cancellation of commands. There were four blocks in the cue-based experiment that prompted the subject for a wink to cancel the execution of the current command after feedback occurrence in each trial, and the other four blocks did not require a wink in the feedback phase. Thus, the TPR was calculated by dividing the number of winks identified in the four blocks that required a wink in the feedback phase by the total number of trials in those four blocks. The FPR was calculated by dividing the number of winks identified in the four blocks that did not require a wink during the feedback phase by the total number of trials in the four blocks. The results in Table 1 showed that the EOG-based switch resulted in a very low FAR with average 0.01 event per minute for all subjects which meant that the switch had good stability and reliability. Subjects were able to use the switch by a triple blink to stop the flicker of buttons in idle state to reduce visual fatigue. The detection of a wink in the feedback phase resulted in an average TPR of 93.54% and FPR of 0.46%, which indicated the capability of using a wink to cancel the command. The efficient detection of the wink in the feedback stage made it more convenient and effective for the robotic arm control. As for SSVEP-based BCI, the results in Table 2 showed that the proposed system worked well in robotic arm control and acquired an average accuracy of 92.09% and the average ITR was 35.98 bit/min. Therefore, the results of the cue-based experiment illustrated the potential of the hybrid BCI to perform complex tasks in practical applications.

**Table 1:** Results of EOG in cue-based experiment.

**Subject**	**Triple blink FAR (event/min)**	**Wink TPR (%)**	**Wink FPR (%)**
S1	0	95.56	0
S2	0	95	0
S3	0	95	1.67
S4	0	100	0
S5	0	96.67	0
S6	0.068	96.67	0
S7	0	96.67	1.67
S8	0.066	93.33	0
S9	0	95	1.67
S10	0	86.67	0
S11	0	78.33	0
Mean ± SD	0.01 ± 0.03	93.54 ± 6.00	0.46 ± 0.78

**Table 2:** Results of SSVEP in cue-based experiment.

**Subject**	**Accuracy (%)**	**ITR (bit/min)**
S1	96.67	39.56
S2	100	42.62
S3	86.67	31.19
S4	93.33	36.57
S5	100	42.62
S6	85.83	30.32
S7	100	42.62
S8	83.78	28.91
S9	97.5	40.33
S10	88.33	33.44
S11	80.83	27.61
Mean ± SD	92.09 ± 7.20	35.98 ± 5.88

**Figure 7:**
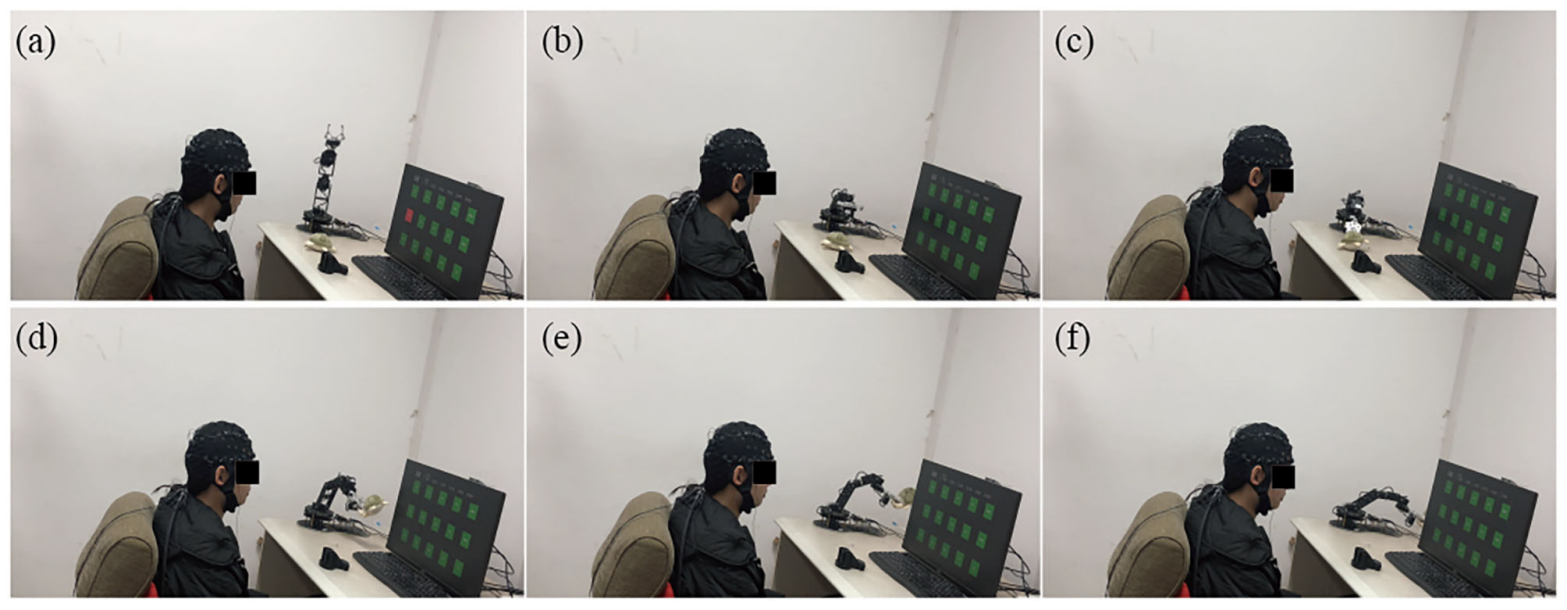
The process of operating the robotic arm to grasp, lift, and move a target object from **(a–f)**.

After the cue-based experiment, subjects were familiar with the procedures of the proposed hybrid BCI, and then they were required to asynchronously utilize the proposed system to operate the robotic arm to perform a complicated task three times by grasping, lifting, and moving a target object (i.e., a little doll) from the initial position to a specific location. Figure 7 showed the process of controlling the robotic arm to complete the specified actions. In the experiment of self-paced operation of the robotic arm, all subjects succeeded in asynchronously grasping and moving the target object from the initial position to the specific location by directly controlling the robotic arm through the hybrid BCI system. To evaluate the efficiency of the hybrid BCI in performing the complex tasks, we recorded the completed time and total number of commands of each subject in the operations of the robotic arm. Table 3 showed the results of the asynchronous experiment for operating the robotic arm through the hybrid EOG-SSVEP-based BCI. Since it was a complicated task for subjects to conduct, the numbers of commands and time required to complete the task were different for each subject, which were related to lots of factors, such as the classification accuracy, the focus and concentration on the task, the planning and grasp strategy, and the proficiency in the robotic arm operation.

**Table 3:** Results of asynchronous robotic arm operation.

**Subject**	**Total number of commands**	**Completion time (s)**
S1	71	394.26
S2	51	284.14
S3	83	473.53
S4	62	349.02
S5	55	305.37
S6	104	586.25
S7	36	197.54
S8	66	371.79
S9	58	324.27
S10	81	474.47
S11	89	500.04
Mean ± SD	68.73 ± 19.38	387.33 ± 112.43

## Discussion

This study attempted to realize an asynchronous hybrid BCI for robotic arm control through the combination of an EOG and SSVEP signals in BCI. Compared with synchronous BCI, the proposed system used EOG-based switch to deactivate the flash to rest or activate the flash to operate the robotic arm whenever they wanted which made the system more flexible and convenient. Additionally, EOG-based timely cancel command allowed users to control the robotic arm to complete a complicated task with a series of actions more effectively. Previous studies which proposed asynchronous SSVEP-based BCI mainly used a conventional threshold method to distinguish the control state from idle state (Cheng et al., 2002; Pfurtscheller et al., 2010b). In these studies, the buttons in the GUI continued to flash from the beginning of the experiment even when subjects were in idle state, which was easy to cause visual fatigue. Several researches designed novel methods to improve the performance of the asynchronous SSVEP-based BCI. Pan et al. proposed asynchronous SSVEP-based brain switches using a pseudo-key-based approach to improve the discrimination between control and idle states (Pan et al., 2013). Pfurtscheller et al. used an MI-based brain switch to achieve self-paced operation of an SSVEP-based orthosis control system (Pfurtscheller et al., 2010b). Tomita et al. proposed of a bimodal BCI using simultaneously NIRS and EEG signals to estimate whether the subject is in idle or active mode (Tomita et al., 2014).

In this study, we chose EOG as the switch signals to either activate or deactivate the flash of the SSVEP-based interface for asynchronous operation of the robotic arm based on the intention of subjects. EOG-based switch in asynchronous SSVEP-based BCI did not need for stimulus in idle state when compared with asynchronous system used the threshold criteria. No stimulus in idle state help relieve fatigue. Moreover, compared with MI-based brain switch and the use of fNIRS signals, the EOG-based switch has the advantage of short response time and high SNR which makes it accurately distinguish the control state from idle state to decrease the FAR in the potential applications. But EOG-based switch also has its limitations. For the experiments lasting for a long time, the major challenge is that subjects may confuse the intended and unintended eye blinks when they get fatigue. Therefore, there is a need for future work to design a simpler and special switch mode, and the improvement of detection algorithm for different eye movements is also helpful. Furthermore, the present study used the SSVEP-based BCI to select specific actions performed by the robotic arm. SSVEP has the advantage of less training and relative high SNR, but the challenge for SSVEP is that it is easy to cause fatigue. In order to reduce user fatigue, we set the buttons to flash between green and blue under black background.

**Figure 8:**
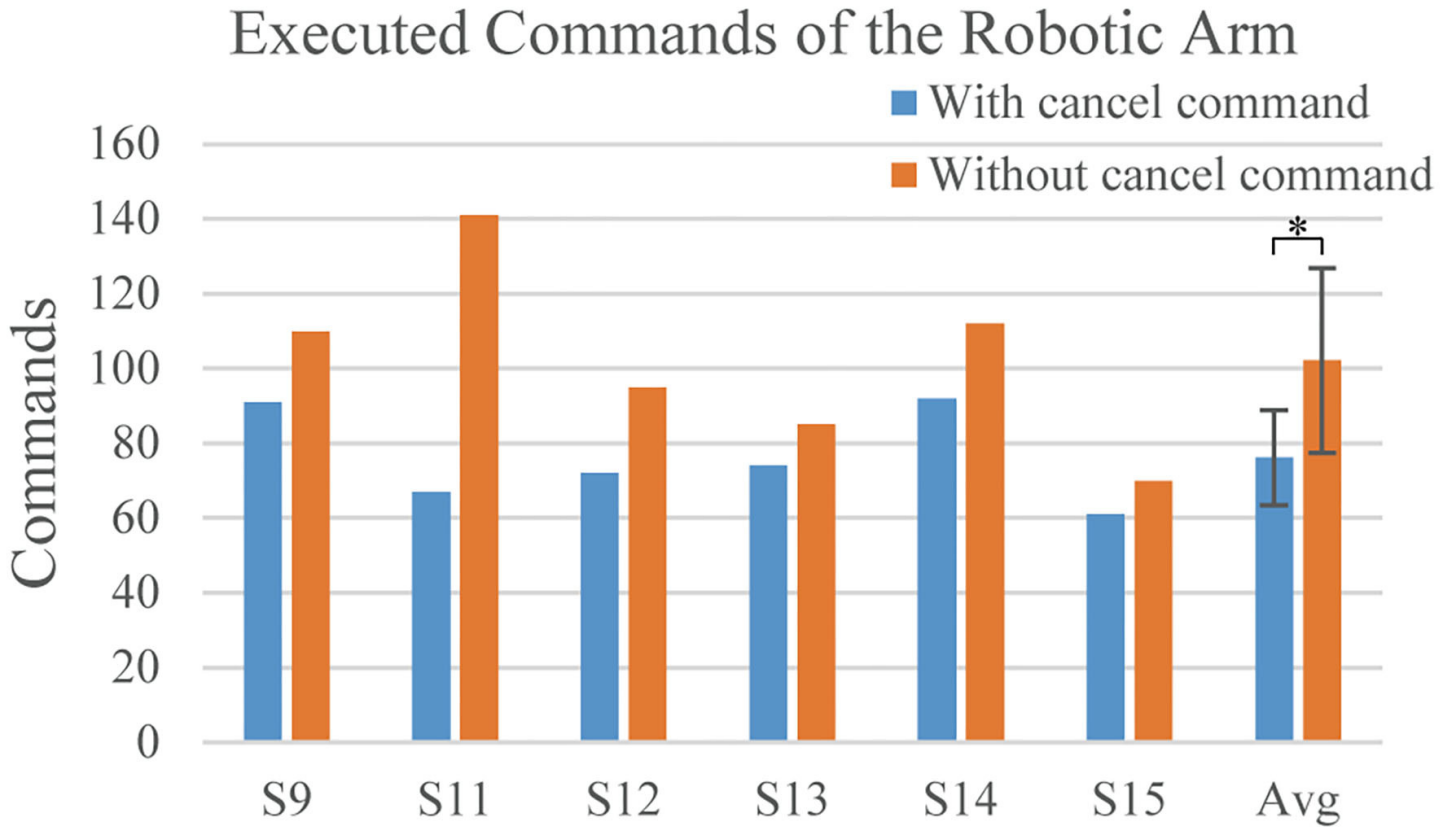
The number of commands executed by the robotic arm for each subject in the grasping task with (blue bar) and without (orange bar) the use of a wink to cancel the feedback command. Avg indicated the average result of all subject. The error bar indicated the standard deviation. We used the paired *t*-test. *indicated the *p* < 0.05.

Additionally, the proposed system allowed subjects to timely cancel the command in feedback phase by a wink to effectively operate the robotic arm to complete a series of grasping, moving, and lifting actions. We implemented a comparable experiment to evaluate the effectiveness of using a wink to cancel command. Six subjects participated in the comparable experiment. Subject S9 and S11 also participated in the previous cue-based experiment. After conducting the calibration process to obtain the thresholds required for EOG detection, each subject was trained to be familiar with using the hybrid SSVEP-based BCI system to perform the grasping task described in the section “Asynchronous Robotic Arm Operation.” And then each subject was asked to perform the grasping tasks with and without the capability of using a wink to cancel the feedback commands three times each. Considering that the execution sequence of tasks might influence the completion of the task, three subjects conducted the grasping tasks with a wink to cancel commands firstly, then without a wink to cancel commands, while the other subjects performed the grasping tasks in reverse sequence. The average number of commands executed by the robotic arm during the grasping task with and without a wink to cancel the commands for each subject was shown in Figure 8. The results indicated that using a wink to cancel the inappropriate commands in the grasping task significantly declined the number of commands executed by the robotic arm. Therefore, the wink-based cancel command helped to improve the effectiveness of robotic arm control.

**Figure 9:**
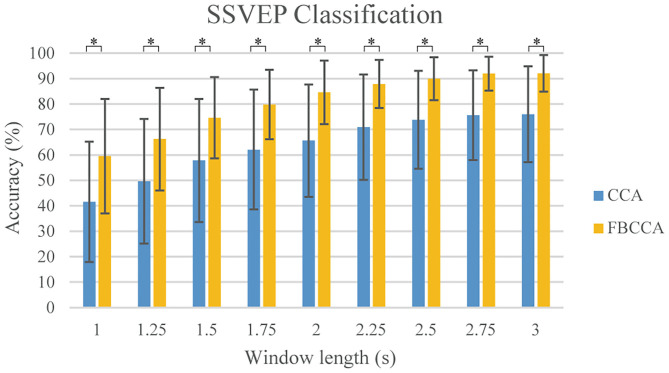
The averaged classification accuracy for 11 subjects under different window length in the cue-based experiment. The blue bar shows the results of CCA and the yellow bar shows the results of FBCCA. Error bars are standard deviations. The asterisk indicates 1% significance level between CCA and FBCCA methods (*t*-test).

**Figure 10:**
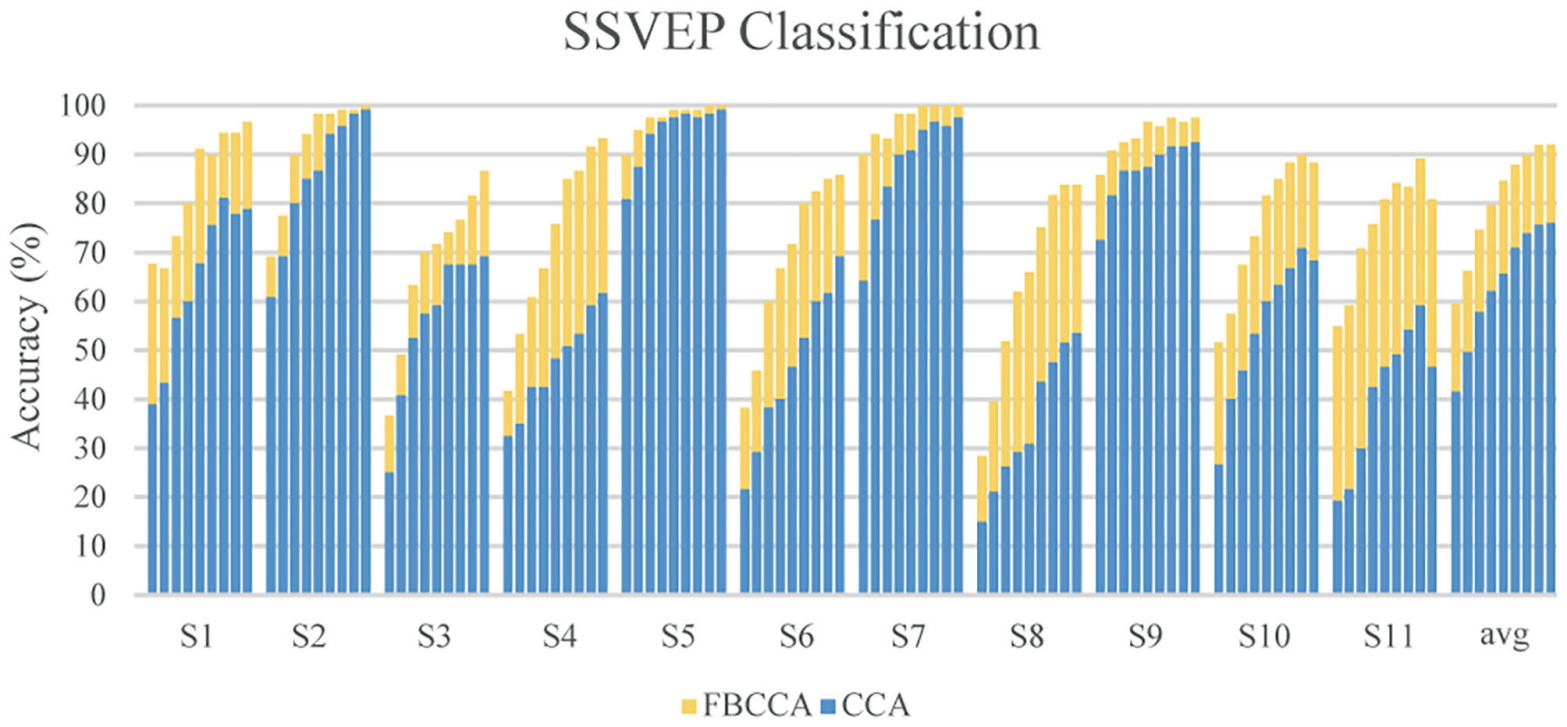
Individual classification accuracy of CCA and FBCCA in the cue-based experiment. Nine bars in a subject indicated nine window length from 1 s **(Left)** to 3 s **(Right)** at a step of 0.25 s. The blue bar shows the results of CCA and the yellow bar shows the results of FBCCA.

As for the classification of the SSVEP-based BCI, we used the FBCCA method for classification, and we compared the classification results of the FBCCA and CCA methods in different window length which was shown in Figure 9. The statistical results revealed that the classification accuracy of the FBCCA was significantly better than the results of the CCA at each window lengths (*p* < 0.01). For the same length of stimulation time, the classification accuracy of FBCCA is better than CCA due to the use of harmonic frequencies information (Chen et al., 2015a,b). Figure 10 showed the individual classification accuracy of CCA and FBCCA methods in the cue-based experiment. The results showed that FBCCA outperformed CCA in each window length for all subjects, especially for those subjects with lower classification accuracy in CCA. However, the flash of buttons in GUI is still easy to cause user fatigue. Recently, several studies attempted to flash the buttons at high frequency to reduce user fatigue (Allison et al., 2010; Diez et al., 2011). Furthermore, several studies showed that BCI combined with technologies like computer vision and deep learning also improve the performance of BCI and reduce the workload of users (Tayeb et al., 2018; Chen et al., 2019). The combination of the proposed asynchronous hybrid BCI and new technologies as future research direction will make the BCI more convenience and user friendly.

## Conclusion

This paper proposed a hybrid BCI which combined SSVEP-based BCI and an EOG-based switch for asynchronous control of the robotic arm. To decrease the FPR in asynchronous BCI, we designed the EOG-based switch to turn on and off the stimulus and a cancel command to effectively accomplish complex tasks. Two online experiments verified the feasibility of the subjects to use the EOG-based switch by a triple blink to activate or deactivate the flash of the SSVEP-based BCI which was used to select the control commands for the operation of the robotic arm to complete a series of complicated movements. And subjects were allowed to timely cancel the current command in feedback phase for more effective control of the robotic arm. All subjects succeeded in asynchronously operating the robotic arm to grasp, lift, and move a target object from the initial position to a specific location. The experimental results suggested that effective combination of EOG and SSVEP signals was able to realize an asynchronous hybrid BCI which allowed user to directly communicate with the external environment based on their own intention.

## Data Availability Statement

The raw data supporting the conclusions of this article are available on request to the corresponding author.

## Ethics Statement

The studies involving human participants were reviewed and approved by Human Subjects Institutional Review Board of Huazhong University of Science and Technology, China. The patients/participants provided their written informed consent to participate in this study. Written informed consent was obtained from the individual(s) for the publication of any potentially identifiable images or data included in this article.

## Author Contributions

YZ, JL, and PL proposed the idea and designed the experiments. YZ conducted the experiments. YZ, YL analyzed the data. YZ and PL wrote the manuscript. JL and PL provided facilities and equipment. All authors contributed to the article and approved the submitted version.

## Conflict of Interest

The authors declare that the research was conducted in the absence of any commercial or financial relationships that could be construed as a potential conflict of interest.
